# Assessment of muscular tone of the tongue using a digital measure spoon in a healthy population: A pilot study

**DOI:** 10.1371/journal.pone.0245901

**Published:** 2021-02-18

**Authors:** Laura Rodríguez-Alcalá, Juan Martín-Lagos Martínez, Carlos O´Connor-Reina, Guillermo Plaza

**Affiliations:** 1 Department of Otorhinolaryngology, Hospital Universitario Clínico San Cecilio, Granada, Spain; 2 Department of Otorhinolaryngology, Hospital Quirón Marbella, Marbella, Spain; 3 Department of Otorhinolaryngology, Hospital Quirón Campo de Gibraltar, Marbella, Spain; 4 Department of Otorhinolaryngology, Hospital Universitario Fuenlabrada, Universidad Rey Juan Carlos, Madrid, Spain; 5 Department of Otorhinolaryngology, Hospital Sanitas La Zarzuela, Madrid, Spain; AUSL della Romagna, ITALY

## Abstract

The study of the muscles of the tongue forms part of a basic evaluation of upper airway function that includes swallowing, speaking and chewing. It is important because the upper airway presents a region of collapse during sleep. Through the action of the dilator muscles, mainly the genioglossus, such collapse can be prevented. In this study, we present a simple tool that can be used to measure the strength of the tongue. This tool may provide an easy way to measure tongue function and allow a simple evaluation of pathologies that affect the tone of the tongue. We have carried out 20 tongue strength measurements using the Tongue Digital Spoon (TDS) in a healthy adult population, using the Iowa Oral Performance Instrument (IOPI) as the gold standard. To validate the procedure, we performed replicate measurements on 20 individuals aged 20–70 years. We found a mean TDS measurement of 115.99 g/cm^2^ in young subjects, 98.47 g/cm^2^ in middle-aged subjects and 84.23 g/cm^2^ in the elderly. There was a significant difference in the measurements between young and elderly participants. There was also a significant correlation between TDS and IOPI measurements (Pearson correlation coefficient, *r* = 0.69, *P* < 0.001). We found the TDS to be a useful tool in daily clinical practice for the measurement of the strength of the tongue in the healthy population. It has potential application in oropharyngeal monitoring and rehabilitation.

## Introduction

The study of the intrinsic and extrinsic muscles of the tongue is basic to understanding the anatomy of the upper airway and to evaluate the muscle functions of swallowing, speaking and chewing [1]. Furthermore, we know that the upper airway presents a region of collapse during sleep. Through the action of the dilator muscles, mainly the genioglossus, such collapse can be prevented [2].

Several studies have focused on the strength of the tongue and its role in the pathophysiology of dysphagia and speech disorders secondary to a central pathology [3]. In these studies, measurements of tongue strength relied upon subjective judgement of the force applied by the tongue versus the resistance provided by a speech therapist holding a tongue depressor [3]. In the past, the reliability of these studies has been questioned due to the subjective variability introduced by the different examiners [4].

Given this limitation, tools have been designed that quantify the strength and resistance of the tongue in a more objective way [4–7]. Furthermore, the differences in the strength and resistance of the tongue in both sexes of a healthy population and in different age ranges have been studied [5]. In the study by Youmans and Stierwalt [4], men had significantly higher peak isometric pressures than women, and younger patients had significantly higher peak pressures than older people.

Several authors have determined that the Iowa Oral Performance Instrument (IOPI) is the most widely used tool for measuring tongue strength [8]. The systematic review and meta-analysis published in 2013 by Adams et al. [9] examined the evidence for the use of the IOPI in 38 studies to measure tongue strength and endurance, concluding that the IOPI is a validated measurement device but is not affordable to all patients and physicians due economical restrains.

We have modified an existing instrument so that it is able to provide a quantitative measurement of lingual strength, and named it the Tongue Digital Spoon (TDS). Our hypothesis is that a simple and more cost-effective tool to measure tongue strength might be useful for those professionals who do not have the IOPI in their work centres. The primary purpose of this study was to assess its reliability and comparability for quantitative clinical tests of oral function. It might also become useful for patients to test themselves during oropharyngeal rehabilitation.

## Material and methods

**Fig 1:**
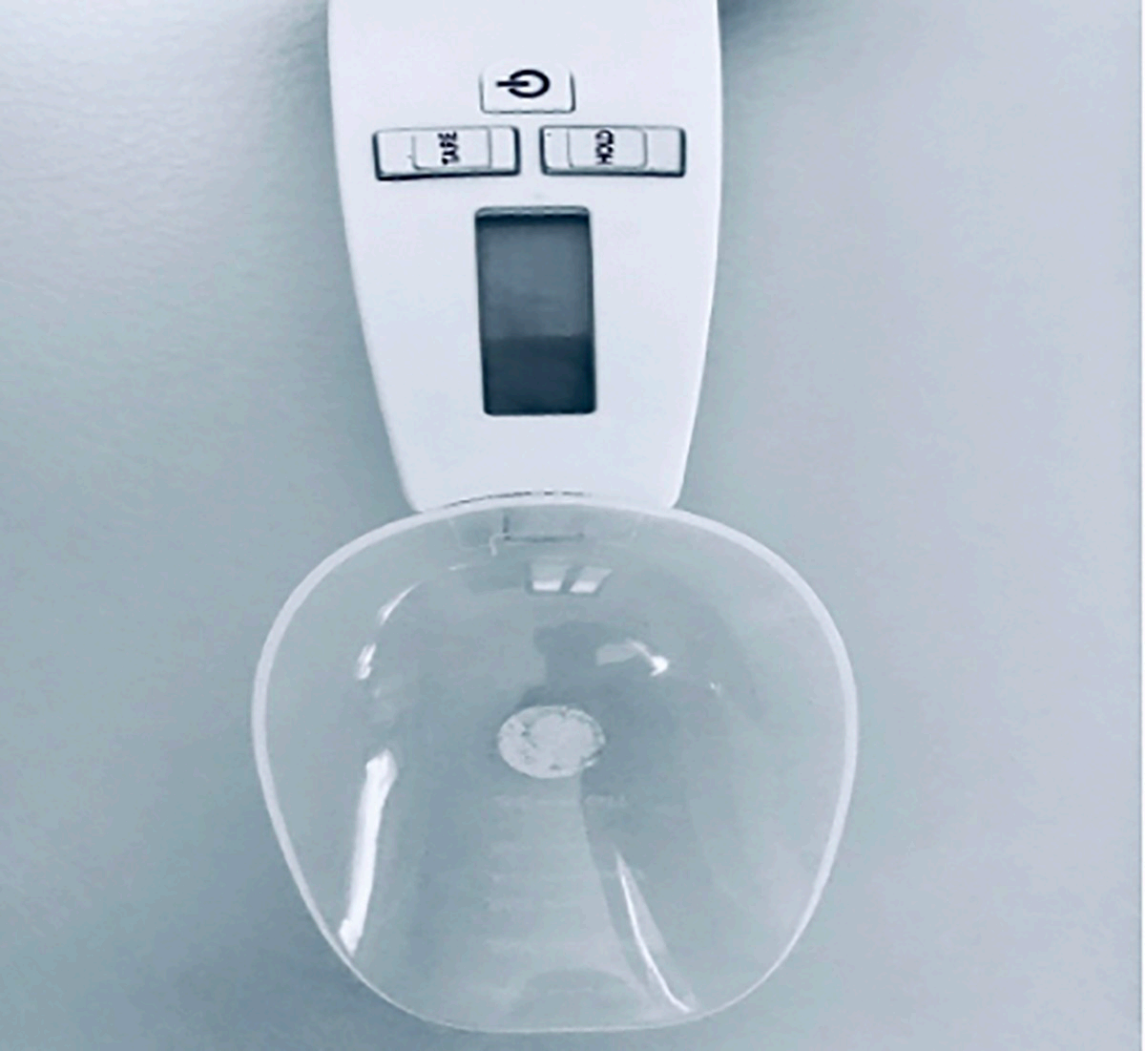
Digital spoon model showing “On/Off” button, “TARE” and “HOLD” buttons and spoon bowl.

**Fig 2:**
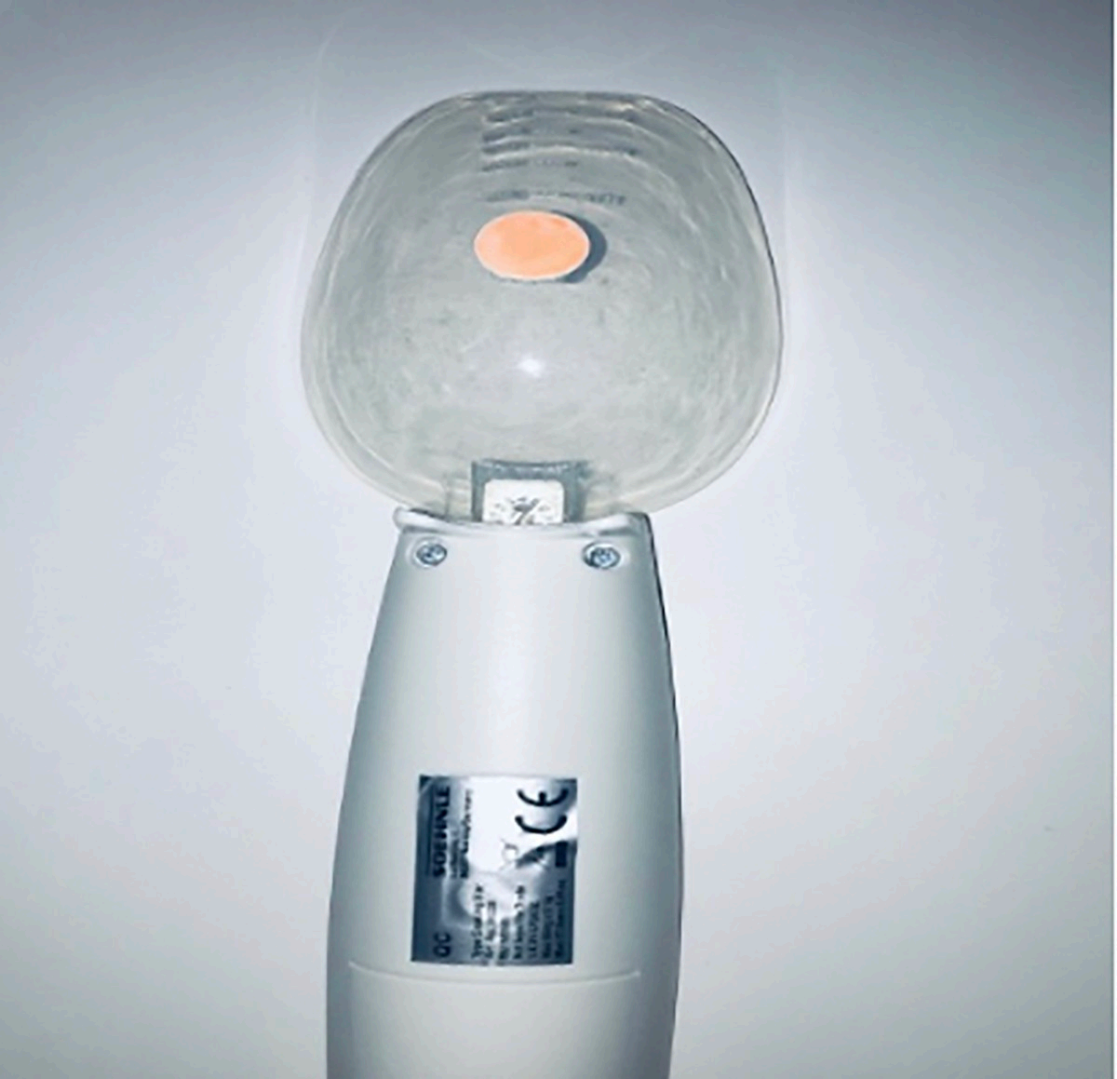
Back of the digital spoon with the 1-cm^2^ sticker placed in the middle (point of maximum support) to take measurements.

**Fig 3:**
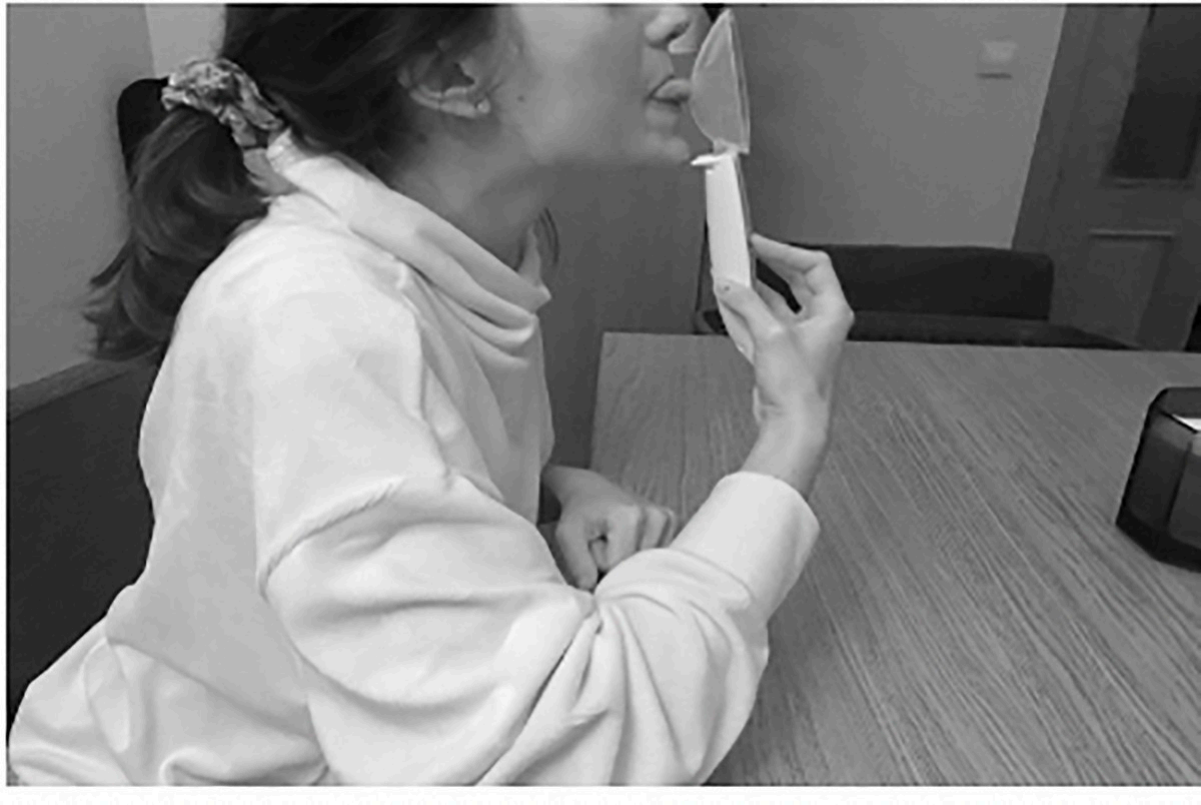
Tongue tone measurement technique with the Tongue Digital Spoon (TDS).

A digital spoon is a kitchen tool used to estimate the weight of food. To develop the TDS, we used the Soehnle Cooking Star Digital Measuring Spoon with graduation from 0.1 g to 500 g *(ID ID20005876833)*. It is a handheld instrument with a spoon that can be found on online shopping platforms. It consists of a handle where the “tare” and “hold” buttons are located. Pressing the “hold” button allows us to obtain the highest tared value, equivalent to the IOPI peak pressure (Figs 1 and 2). To carry out the measurements, the spoon is inverted and a 1 cm^2^ circular sticker is placed on the back, obtaining a marked circumference. To measure tongue strength, the subject holds the spoon by the handle and, with their elbow resting on a flat surface, brings the spoon closer to the tongue with an elbow angle of approximately 30°. The subject must tare the device by pressing the “hold” key, marking 0.0 g. Then, the subject presses on the marked circumference with the tip of their tongue. Once done, with the index finger of the hand that holds the handle, the subject presses the “hold” button. This test is performed entirely by the subject to avoid movements on the spoon that may interfere with the results (Fig 3).

The individuals pictured in Fig 3, S1–S3 Videos have provided written informed consent (as outlined in PLOS consent form) to publish their image alongside the manuscript.

Our local Ethics Committee approved the study (AWGAPN-2019-01), and all participants gave written informed consent. We have selected 20 healthy subjects, aged 20–70 years. The sample was obtained from health care workers of Hospital Universitario Clínico San Cecilio (Granada, Spain) that were recruited during the months of March-August 2020. With the aim of obtaining oral healthy subjects without dysfunction of lingual motility, several exclusion criteria were adopted (Table 1).

**Table 1:** Exclusion criteria.

• History of cancer or surgery of the mouth or tongue • Obstructive sleep apnoea • Neurological disease • Pregnancy • Body mass index > 30 • Dysphagia (including atypical dysphagia) • Absence of teeth or dental prostheses • Gastro-esophageal syntoms • Presence of pathological frenulum • Previous chemotherapy or local radiotherapy • Upper airway obstruction (septal deviation, adenotonsillar hypertrophy)

A calculation of the sample size (N) has not been carried out since initially the number of subjects to be included was going to be small because it is a new diagnostic technique. We have not considered calculating the power (P) of the study since the sample size is small and surely by increasing the N in subsequent studies, we will obtain a P that allows us to detect the desired results. However, our sample can be considered representative of a larger healthy population.

All participants were evaluated by an otolaryngologist and speech therapist for an anatomical and functional exploration of the upper airway. We performed tongue strength measurements with the TDS and with the IOPI. Measurements were performed three times using each instrument on each person and the highest value was recorded.

Data were analysed with IBM SPSS Statistics Base 22.0 software (Armonk, NY). Baseline characteristics, age and sex, were analysed independently in each of the study groups. The Mann–Whitney *U* test was used for statistical analysis of variables between the groups. Quantitative values were expressed as mean and standard deviation (SD). The Pearson correlation coefficient *(r)* test was performed for the main quantitative variables of the study with a value of *P* < 0.05 being regarded as significant.

## Results

In a series of 20 healthy people (12 men and 8 women), we carried out measurements with the TDS. We calculated the mean and SD of the values obtained in subjects grouped by different age ranges (young, 20–39 years; middle-aged, 40–60 years; and elderly > 60 years) (Table 2) and sex (Table 3). The mean values with the TDS were 115.99 ± 17.47 g/cm^2^ in the young group, 98.47 ± 11.51 g/cm^2^ in the middle-aged group, and 84.23 ± 14.53 g/cm^2^ in the elderly group. There were statistically significant differences in the mean TDS values between the young and elderly groups (*P* = 0.02) and the middle-aged and elderly groups (*P* = 0.05). There were also differences in the TDS values between women and men: 114.61 ± 10.32 and 119.87 ± 11.51, respectively (*P* = 0.05).

**Table 2:** Mean values of the strength of the tongue (g/cm^2^) measured with the Tongue Digital Spoon (TDS) in different age groups.

	Mean	SD	N	*P*
**Young (20–40 years)**	115.99	17.47	7	Young vs elderly
				*P* = 0.02*
**Middle-aged (40–60 years)**	98.47	11.51	9	Young vs middle-aged
				*P* = 0.07
**Elderly (> 60 years)**	84.23	14.53	4	Middle-aged vs elderly
				*P* = 0.05*

SD = standard deviation. *P* < 0.05 (Mann–Whitney *U* test).

**Table 3:** Mean values of the strength of the tongue (g/cm^2^) in women and men measured with the TDS.

	Mean	SD	N	*P*
**Female**	114.61	10.32	8	0.05*
**Male**	119.87	11.51	12	

(Mann–Whitney *U* test). SD = standard deviation.

**Fig 4:**
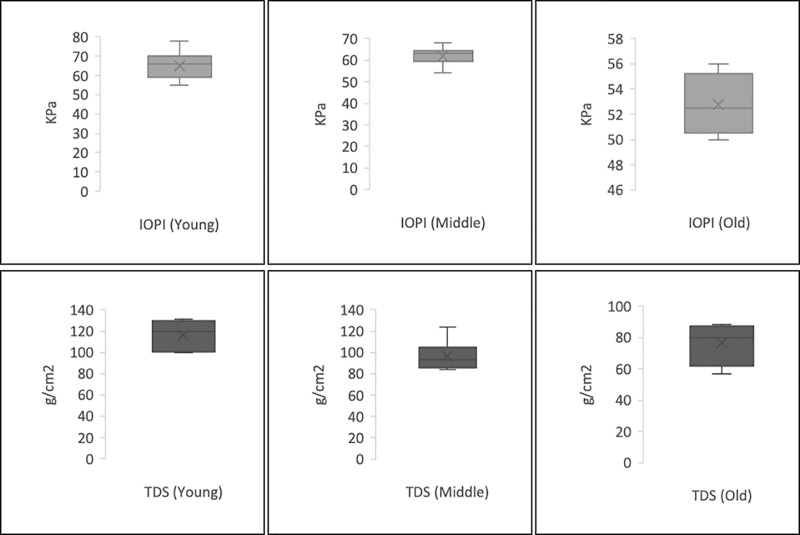
Distributions of measurements of methods for assessment maximum tongue pressure: TDS (g/cm^2^) and IOPI (kPa) in each group.

We compared our TDS results with those we obtained using the IOPI with the same participants (Table 4, Fig 4). The mean values with the IOPI were 65 ± 14.3 in the young group, 64 ± 12.6 in the middle-aged group, and 52 ± 13.8 in the elderly group. There were statistically significant differences in the mean IOPI values between the young and elderly groups (*P* = 0.03) and the middle-aged and elderly groups (*P* = 0.04). There were also differences in the IOPI values between women and men: 54 ± 11.8 and 65 ± 12.2 (*P* = 0.04), respectively.

**Table 4:** Mean values of the strength of the tongue (anterior tongue strength in kPa) measured with the IOPI in different age groups.

	Mean	SD	N	*P*
Young (20–40 years)	65	14.3	7	Young vs middle-aged
				*P* = 0.03*
Middle-aged (40–60 years)	64	12.6	9	Young vs middle-aged
				*P* = 0.22
Elderly (> 60 years)	52	13.8	4	Middle vs elderly
				*P* = 0.04*

SD = standard deviation. (Mann–Whitney *U* test). SD = standard deviation.

**Fig 5:**
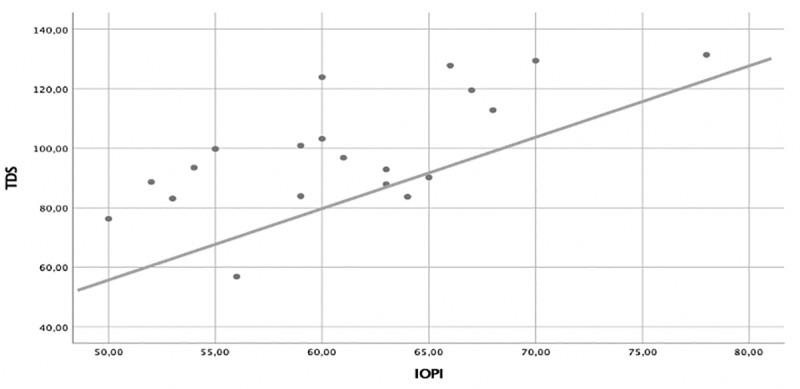
A significant positive correlation was observed between the Iowa Oral Performance Instrument (IOPI) and the TDS in the measurement of the tongue strength (*r* = 0.69, *P* < 0.01).

Finally, we searched for a correlation between the strength of the tongue measurements taken on both instruments, finding a significant correlation coefficient, *r* = 0.69. *P* < 0.001 (Fig 5).

## Discussion

In the present work, we compared the ability of two instruments to measure tongue strength in healthy people. We used the IOPI values as a reference [8] as numerous studies are based on IOPI measurements [3, 4, 9–14]. We compared these values with measurements taken with a new instrument, the TDS. To our knowledge, this is the first study to analyse the efficacy of this type of kitchen tool when used to measure tongue strength.

Our development of the TDS technique was based on the study by Kim et al. [15]. In that study, to measure the maximal isometric tongue protrusion force participants sat upright with the tongue extended 0.5–1 cm beyond the teeth to touch a piston. The participants were instructed to push their tongue forward against the piston with the maximum possible force for 2–5 seconds. The results were measured in Newtons. With the TDS, in addition to protruding the tongue and exerting maximum pressure on the spoon, the patient must press the “hold” button. To increase the reliability of the test, we performed 3–4 repetitions of each measurement. The first 2–3 measurements were used for familiarisation with the instrument, and we chose the highest one. The results were measured in g/cm^2^.

To measure the strength of the tongue, other studies have used measurement instruments similar to the IOPI. For example, in the study by Miura et al. [6], the authors measured the maximum pressure of the tongue using a tongue pressure device. The device consisted of a probe with a balloon-type pressure sensor and a plastic cylinder to fix the location of the probe during measurement. The median maximum tongue pressure was 42.6 kPa (IQR: 36.1–46.4 kPa). As in our study, significant differences were observed between men and women with respect to the maximum pressure of the tongue (*P* = 0.02).

Another instrument is the JMS tongue pressure measurement device (JMS) (TPM-01; JMS Co., Ltd. Osaka, Japan) Maximum tongue pressure measurements using the IOPI and the JMS were significantly correlated [10], although only a small number of studies have focused on the relationship between these two instruments. Yoshikawa et al. [16] reported higher values with the IOPI compared to the JMS prototype, despite a significant correlation between the devices. Although the average discrepancy between the two methods was relatively low (+8.99 kPa), it remains unclear whether distinguishing such discrepancies is of clinical interest. A Spearman correlation analysis also indicated a moderate correlation between the IOPI and the JMS (*r* = 0.39; *P* < 0.049). In our study, although the values were expressed with different measures (g/cm^2^ and kPa), we also found a significant correlation between the TDS and the IOPI (*r* = 0.69; *P* < 0.01).

In the study by Paris-Alemany et al., the authors found that the position of the tongue in relation to the craniomandibular region could affect oral function [17]. Using the IOPI in three different cranio-cervical positions: neutral head position (NHP), anterior head translation or forward head position (FHP) and posterior head translation or retracted head position (RHP), tongue resistance measurements showed statistically significant differences for FHP (*P* = 0.001), NHP (*P* = 0.00) and RHP (*P* = 0.007). The cranio-cervical position influences the strength of the tongue, especially in the anterior and middle areas of the tongue. In our study, we performed all measurements with the IOPI and the TDS with subjects in a NHP for studies of the strength of the anterior area of the tongue.

In the studies by Park et al. [18] and Van den Steen et al. [19], the effect of exercise on increasing tongue strength in healthy young adults and healthy older adults was examined. The ages were between 21–28 years, 28–65 years and > 65 years in each of the studies, separated into three groups. After training, the tongue strength measures increased significantly in all groups. However, there were no significant differences in strength gains between the age groups. In our work, we found significant differences in the baseline measurements using both the TDS and the IOPI between young and elderly groups and middle-aged and elderly groups; however, no differences were found between the young and the middle-aged groups.

With our study, we aimed to provide a readily available instrument that could be used for the measurement of tongue force in clinical or research practice. We compared oral function measurement with both devices and examined the relationship between the TDS measurements and independent factors such as age and sex in healthy adults. Consequently, we observed that the strength of the tongue measured with the TDS correlated significantly with the strength of the tongue measured with the IOPI device.

Several authors have used IOPI to monitor the effect of tongue exercises on tongue strength, both in healthy adults [20, 21], patients suffering dysphagia [22] or sleep disordered breathing [23–25]. The TDS could be an invaluable tool to allow self-measured monitoring of myofunctional therapy in patients with sleep-disordered breathing, in a similar way that IOPI tests are used to measure improvements in the apnoea–hypopnea index [23, 24]. We anticipate this tool will increase adherence to therapy as it provides information about the benefit of the therapy to the patient. If the scores obtained after performing exercises, several days are higher comparing from the beginning, its mean that the exercises are being performed properly. This is especially important during the COVID-19 pandemic, where medical visits are restricted and all in-home procedures are encouraged. As conventional tongue strength measurement devices are expensive and normally restricted to hospital and medical office use, it is likely this tool will be useful at this special time.

The present study has some limitations. First, it is a pilot study with a small sample size. Second, it is based on healthy subjects, excluding patients with lingual hypotonia. Finally, we need to confirm, in a new study, any improvement of the TDS scores after performing oropharyngeal exercises, as has been studied with regard to the IOPI.

## Conclusions

We consider the Tongue Digital Spoon (TDS) a useful instrument to measure the strength of the tongue. Advantages over other measuring instruments are its ease of use as a technique, its low cost and its easy availability worldwide. It has a potential use for oropharyngeal monitoring and rehabilitation.

## Supporting information

S1 Video Tongue tone measurement technique with the Tongue Digital Spoon (TDS) performed by the first author. (MP4)

S2 Video Tongue tone measurement technique with the Tongue Digital Spoon (TDS) performed by the first author. (MP4)

S3 Video Tongue tone measurement technique with the Tongue Digital Spoon (TDS) performed by the first author. (MP4)

S1 File (XLSX)

S2 File (RTF)
