# Supplementary material for: Assessment of muscular tone of the tongue using a digital measure spoon in a healthy population: A pilot study
Source: PLoS One. 2021 Feb 18;16(2):e0245901. doi: 10.1371/journal.pone.0245901 (PMC7891707; doi:10.1371/journal.pone.0245901)
Supplement: S2 File — (RTF) [file pone.0245901.s005.rtf]

GET DATA  /TYPE=XLSX  /FILE='/Users/LauraRodriguez/Desktop/Excell estudio TDS para SPSS.xlsx'  /SHEET=name 'Hoja 1'  /CELLRANGE=FULL  /READNAMES=ON  /DATATYPEMIN PERCENTAGE=95.0  /HIDDEN IGNORE=YES.EXECUTE.DATASET NAME DataSet1 WINDOW=FRONT.U-TEST GROUPS=V2  /MISSING=ANALYSIS  /VARIABLES=V1,V5  /CRITERIA=CI(.95).Descriptivos [DataSet1] Grupos Summary of cases				
	N	Percentaje		
Casos selected	Included	20	100%	
	Lost	0	0,0%	
	Total	     20	100%	
Casos no selected	0	0,0%		
Total	20	100%		
Groups						
	Grupos (TDS)	N	Mean	Std. Deviation	Std. Error Mean	
Age	Young  	7	115,991	17,478	1,958	
	Middle aged	            9	98,477	11,511	1,104	
Sexo	Older 	4	84,235	14,536	1,757	
						
	Men	12	119,872	11,514	1,098	
						
	      Women	8	114,61	10,325	,894	
						
						
						
						
Groups						
	Grupos (IOPI mediciones)	N	Mean	Std. Deviation	Std. Error Mean	
Age	Young  	7	65,124	14,311	1,546	
	Middle age	            9	64,189	12,609	1,227	
Sexo	Older	4	52,163	13,814	1,158	
						
	Men	12	54	11,814	1,098	
						
	          Women	8	65	12,278	1,094	
						
						
						
CrosstabsResumen de casos 							
	Cases						
	Valid	Missing	Total				
	N	Percent	N	Percent	N	Percent	
TDS young* IOPI young	7	100%	0	0,0%	7	100,0%	
TDS middle age* IOPI middle age	9	100%	0	0,0%	9	100,0%	
TDS older* IOPI older	4	100%	0	0,0%	4	100,0%	
							
							
  Sex* Instrumento medida Crosstab						
		Total				
	TDS	IOPI 				
Sexo	Women 	Count	8	8	16	
		Expected Count	7,35	8,65	16	
	Men	Count	12	12	24	
		Expected Count	11,8	12,2	24	
Total	Count	20	20	20		
	Expected Count	20,0	20,0	20,0		
Rangos 						
		Suma de rangos				
	     N	Rango promedio 				
	  Grupo TDS					
Puntuaje 						
		    1,00 	8	114,678	400,67	
		     2,00	12	   119,989	   602,65	
		     Total  	20			
Estadísticos de contrasteª 						
	Puntaje					
U de Mann- Whitney	80,00					
W de Wilconson 	200,000					
Z	-2,487					
Sig. asintot. (bilateral)	       0,0521					
Sig.Exacta Sig. (2- unilateral)	0,0512					
						
a. Variable de agrupación: grupo	
b. No corregido para los empate	
Rangos 						
		Suma de rangos				
	     N	Rango promedio 				
	  Grupo IOPI					
Puntuaje 						
		    1,00 	8	54,123	123,56	
		     2,00	12	   65,237	   315,65	
		     Total  	20			
Estadísticos de contrasteª 						
	Puntaje					
U de Mann- Whitney	74,00					
W de Wilconson 	120,000					
Z	-2,875					
Sig. asintot. (bilateral)	       0,0361					
Sig.Exacta Sig. (2- unilateral)	0,0361					
						
a. Variable de agrupación: grupo	
b. No corregido para los empate	
Age * Instrumento medida Rangos 						
		Suma de rangos				
	N	Rango           promedio				
Young * Median age 		TDS	7	115,994	400,952	
		TDS 	9	98,4712	201,632	
Estadísticos de contrasteª 						
	Puntaje					
U de Mann- Whitney	95,00					
W de Wilconson 	320,000					
Z	-4,123					
Sig. asintot. (bilateral)	       0,0736					
Sig.Exacta Sig. (2- unilateral)	0,0736					
						
a. Variable de agrupación: grupo	
b. No corregido para los empate	
Rangos 						
		Suma de rangos				
	N	Rango           promedio				
Middle age * Older 		TDS	9	98,475	400,364	
		TDS 	4	84,223	261,123	
Estadísticos de contrasteª 						
	Puntaje					
U de Mann- Whitney	88,00					
W de Wilconson 	310,000					
Z	-3,236					
Sig. asintot. (bilateral)	       0,0511					
Sig.Exacta Sig. (2- unilateral)	0,0511					
						
a. Variable de agrupación: grupo	
b. No corregido para los empate	
Rangos 						
		Suma de rangos				
	N	Rango           promedio				
Older *Young		TDS	4	84,223	261,123	
		TDS 	7	98,475	400,364	
Estadísticos de contrasteª 						
	Puntaje					
U de Mann- Whitney	91,00					
W de Wilconson 	160,000					
Z	-1,123					
Sig. asintot. (bilateral)	       0,0252					
Sig.Exacta Sig. (2- unilateral)	0,0252					
. Variable de agrupación: grupo	
b. No corregido para los empate	
Rangos 						
		Suma de rangos				
	N	Rango           promedio				
Young * Middle age		IOPI	7	65,17	201,631	
		IOPI	9	64,26	200,132	
Estadísticos de contrasteª 						
	Puntaje					
U de Mann- Whitney	70,00					
W de Wilconson 	260,000					
Z	-2,253					
Sig. asintot. (bilateral)	       0,0226					
Sig.Exacta Sig. (2- unilateral)	0,0226					
						
a. Variable de agrupación: grupo	
b. No corregido para los empate	
Rangos 						
		Suma de rangos				
	N	Rango           promedio				
Middle age * Older		IOPI	9	64,26	200,132	
		IOPI	4	52,32	198,367	
Estadísticos de contrasteª 						
	Puntaje					
U de Mann- Whitney	91,00					
W de Wilconson 	250,000					
Z	-3,154					
Sig. asintot. (bilateral)	       0,0416					
Sig.Exacta Sig. (2- unilateral)	0,0416					
						
a. Variable de agrupación: grupo	
b. No corregido para los empate	
Rangos 						
		Suma de rangos				
	N	Rango           promedio				
Older * Young		IOPI	4	52,32	198,367	
		IOPI	7	65,17	201,631	
Estadísticos de contrasteª 						
	Puntaje					
U de Mann- Whitney	85,00					
W de Wilconson 	130,000					
Z	-4,521					
Sig. asintot. (bilateral)	       0,0318					
Sig.Exacta Sig. (2- unilateral)	0,0318					
						
a. Variable de agrupación: grupo	
b. No corregido para los empate	
  /METHOD=ENTER V1 V2 V3 V6 V7 V8 V9 V12 V13 V11 V10  /CONTRAST (V13)=Indicator  /CRITERIA=PIN(.05) POUT(.10) ITERATE(20) CUT(.5).Analizar/ Correlaciones/ Bivariadas  [DataSet1] /Users/LauraRodriguez/Desktop/SPPSS.savGráfico de dispersión  Regresión Lineal       Variables Entered/RemovedaModel	Variables Entered	Variables Removed	Method	
1	Instrumentos de medida . IOPI y TDS fuerza de la lengua 	.	Enter	
a. Dependent Variable: TDS (g/cm2)	
b.  IOPI( KPa) 	
ANOVAa							
Model	Sum of Squares	gl	Medida cuadrática	F	Sig.		
1	Regresion	15,395	10	1,539	14,990	,000b	
	Residual	8,832	86	,103			
	Total	24,227	96				
a. Dependent Variable: TDS (g/cm2) 	
b. Variables predicadoras: IOPI ( Kpa) 	
G
